# The Interplay of E-commerce, Resilience and Exports in the Context of COVID-19

**DOI:** 10.1007/s10796-022-10342-w

**Published:** 2022-10-15

**Authors:** Adah-Kole Emmanuel Onjewu, Sundas Hussain, Mohamed Yacine Haddoud

**Affiliations:** 1 Newcastle Business School, Northumbria University, Newcastle upon Tyne, UK; 2 Nottingham Business School, Nottingham Trent University, Nottingham, UK; 3 Faculty of Business and Law, The British University in Dubai, Dubai, UAE

**Keywords:** E-Commerce, Resilience, Direct Exports, Indirect Exports, SMEs, COVID-19

## Abstract

Scholars have extolled e-commerce as a pathway for sustaining firm operations in the unique circumstances of COVID-19. To add crisis time evidence to the body of work, and based on World Bank Enterprise Survey data, this inquiry interrogates 249 small manufacturing firms in Italy by examining the effect of e-commerce on (1) resilience, (2) direct exports and (3) indirect exports. The results show that while e-commerce has a positive impact on direct exports, a negative influence is recorded on indirect exports. Besides, e-commerce significantly increases resilience which, in turn, accelerates direct exports. However, resilience is found to have a trivial influence on indirect exporting. Furthermore, held as a constant, firm size demonstrates a significant and positive effect on direct and indirect exports. These fresh findings prompt implications for understanding the correlates of e-commerce, resilience and export behaviour. Practically, there are salient insights for stakeholders engaged in reviving small manufacturers’ exports for a speedy post COVID-19 recovery in Italy.

## Introduction

E-commerce is the selling of goods and services via online channels (Khan, 2016), and is one of several instruments available in firms’ digital transformation toolbox (Matarazzo et al., 2021). At the firm level, the imperative to digitally transform the sales process is intensified by slumps such as the COVID-19 recession (Rapaccini et al., 2020). Faced with fiscal pressure, firms reconsider and reconfigure their use of digital technologies to fashion new business models for greater creation and appropriation of economic value (Verhoef et al., 2021). In the fashion industry, Gaudenzi et al. (2021) found that e-commerce offers a pathway for firms to evolve in two directions. In the first path, they contend that it bestows resources in the form of a network structure in the supply chain and a service architecture for product variation and delivery responsiveness. Secondly, they reasoned that e-commerce enables the development of capabilities in relationship governance, managing information asymmetry, logistical operations and virtual customer relationship management. In theory, these resources and capabilities typify some of the characteristics of resilience needed by firms to navigate the COVID-19 challenges in terms of agility and elasticity (Rapaccini et al., 2020). Therefore, with digitalisation in mind, there is a current yearning to better understand the mechanism by which e-commerce supports resilience and subsequent firm performance during crisis.

The interest in firm resilience among scholars and the wider public is cyclical and typically amplified by stints of economic hardship (Burger et al., 2017; Haddoud et al., 2022). In periods like the COVID-19 episode, the endeavour to uncover the ingredients for resilience is only eclipsed by attempts to define the attribute in terms that are more conceptually and empirically definitive. Current understanding of resilience is loose possibly because of multidisciplinary interest in the concept in fields including ecology, engineering, governance, and management (Angeler & Allen, 2016). In the latter domain, it [resilience] has been described as the capacity to bounce back from adversity (Luthans, 2002), as well as the ability to rebuild by exploring and enacting new opportunities for bouncing forward (Muñoz et al., 2019). Nevertheless, Holling’s (1973) long-held view of resilience as the amount of disturbance a system can withstand before shifting into an alternative state still provokes reflection for two reasons. First, the emphasis on ‘system disturbance’ permits the isolation and observation of exclusive black swan events impinging on firm performance (Taleb, 2010). Second, ‘shifting into an alternative state’ signals and enables the investigation of point-in-time survivability that is essential for believing that the firm is a going concern. To this end, Gray & Alles (2021: 199) put forward a ‘going concern survivability index’ as the percentage reduction in revenue that the firm can absorb before losing its ability to continue as a going concern. For empirical work, this specification of revenue in relation to survival offers opportunity for a novel operationalisation of firm resilience.

Following the preceding predication, Engert et al. (2022) recently reported the continued growth in the adoption of e-commerce content management systems. E-commerce has proven to be a pathway for cultivating firm resilience in both developed and developing settings during the COVID-19 crisis (Chowdhury et al., 2021), as firms’ performance has been threatened by the fragile environment (Alraja et al., 2022). As a resource, e-commerce has the benefit of enabling firm decision-makers to gather and convert data into actionable knowledge for improving organisational performance (Choi & Lee, 2017). Particularly, when online interactions successfully morph into offline relationships, firms are also able to increase consumers’ repurchasing intention and referral rate (Xiao et al., 2019). However, during a crisis, the precise attraction of e-commerce is that it affords firms the opportunity to enter local and global markets on a low operational budget while increasing customer reach and retention (Kartiwi et al., 2018). The development of such capability is equally true in manufacturing environments as internet use has been determined to stimulate export activity among manufacturers in OECD countries [including Italy] (Bojnec & Fertö, 2009).

To elaborate on exports, Katsikeas et al. (2019) allude to digital technologies such as e-commerce being facilitators of foreign market entry. The stream of literature linking e-commerce to export behaviour is admittedly vast. In one study, Gregory et al. (2007) demonstrated that e-commerce assets directly increase firms’ export intensity. Also, Alavinasab & Taleghani (2016) showed that e-commerce factors including technological assets enhance export performance. Likewise, Hinson & Sorensen (2006) revealed that firms are more likely to export if they engage in some e-commerce activity. Yet, by commission or omission, there is a palpable pattern in research in this area to focus on the utility of e-commerce for direct exports without weighing the impact of the former on indirect exporting undertaken through intermediaries (Rialp-Criado & Komochkova, 2017). This tendency to overlook consideration and measurement of exports in both its direct and indirect forms as urged by Love & Roper (2015) may curtail full understanding of firms’ export performance. Motta (2020) recently stressed the importance of empirically decomposing direct and indirect exporting as these activities deplete firm resources, like labour, in dissimilar ways.

The current interest in export performance over other areas of organisational activity hinges on prior indications that internationalisation is a particularly challenging endeavour for SMEs (Haddoud et al., 2018; Abubakar et al., 2019; Haddoud et al., 2021a). Thus, consistent with the self-selection hypothesis (Monreal-Pérez et al., 2012), there is reason to believe that only the most productive SMEs are likely to export during a crisis such as COVID-19. In this sense, such companies would naturally demonstrate high performance financially and operationally, and exporting will suffice as a more exacting indicator of their capability. This stance diverges from the learning-by-doing perspective which purports that firms will engage in exporting after accumulating sufficient knowledge for participation in export markets (Manjón et al., 2013).

Against this backdrop, the purpose of this study is to isolate and examine the effect of e-commerce activity on resilience in the unique circumstances of the COVID-19 crisis. It is also conceived to investigate the discrete and direct influence of e-commerce and resilience on (1) direct exports and (2) indirect exports to heed earlier appeals by Rialp-Criado & Komochkova (2017) and Motta (2020). The overriding question pondered here is whether e-commerce activity is independently sufficient to generate direct and indirect exporting during the COVID-19 crisis, and the extent to which resilience, if at all, plays a role in these correlations. To this extent, the contribution of this inquiry is fourfold. First, it develops and validates a novel link between e-commerce, resilience and export behaviour as there is limited precedent of these associations being conceptualised or tested. Second, the study advances the measurement and investigation of resilience by examining a relatively underexplored conceptualisation that takes into account the amount of disturbance a system can withstand before shifting into an alternative state (Holling, 1973). As a first step to resilience, there is value in discerning how long firms can withstand shocks before needing to bounce back. The current literature tends to bypass this step to directly address ‘bouncing back’ without considering firms’ endurance. Third, the study gathers crisis time evidence to assess resilience during a period of system disturbance that genuinely portends the likelihood that firms will shift into an alternative state. In this regard, to the best of the authors’ knowledge, this study is one of the first to offer COVID-19 evidence from severely hit Italy. No previous studies have assessed the interplay of e-commerce, resilience, direct and indirect exports in the Italian manufacturing scene using a quantitative design. Fourth, the inherent analysis provides specificity by separating the effects of e-commerce and resilience on direct exports and then indirect exports. This does not only respond to Rialp-Criado & Komochkova (2017) and Motta’s (2020) calls for discerning direct and indirect exporting, but also addresses Lafuente et al. (2019) and Rahman and Mendy’s (2019) plea for further evidence in the resilience-internationalisation nexus.

Pressing forward, the rest of this article is organised as follows: Sect. 2 presents the research context while Sect. 3 conceptualises e-commerce and resilience as predictors of both forms of export. Subsequently, Sect. 4 explains the measurement variables, items and scales before findings are offered in Sect. 5. In Sect. 6, the findings are compared and synthesised with prior literature by way of a discussion. The paper concludes with theoretical contributions, practical implications, and areas for future research in Sect. 7.

## Small and Medium Italian Manufacturers

Italy is the southern European country bordering France, Switzerland and Austria (Jakubowski, 2018). With 60 million inhabitants occupying its 300,000 square kilometres surface area, it is one of the most populous and largest countries on the continent (Berardi et al., 2020). Economically, Italy is an industrialised state and a leading exporter (Kaplinsky & Morris, 2019). In 2019, its gross domestic product exceeded $2 trillion and 31.5% of its outputs were exported (The World Bank, 2021c). To be ‘made in Italy’ signals the cultural expression of Italian craftsmanship especially in fashion, food, furniture, and mechanical engineering (Aiello et al., 2015). In consumers’ minds, the expression evokes an exclusive image of Italian artistry, aesthetics, quality and sophistication through the effect of product-country association (Temperini et al., 2016). Accordingly, researchers interested in the country have examined several antecedents and outcomes in Italian manufacturing including research and development investment (Hall & Oriani, 2006), innovation and profitability (Cozza et al., 2012), manufacturing servitisation (Mastrogiacomo et al., 2017), and the circularity of business practices (Blasi et al., 2021). Of particular consideration in the Italian scene is the exceptionally high proportion of micro and small enterprises in comparison to other industrialised economies (BPI, 2015). Recent figures released by the Italian National Institute of Statistics [ISTAT] show that micro enterprises with 3–9 employees constitute 79.5% of firms in the economy. In turn, small enterprises with 10–49 employees comprise 18.2%, while medium-sized [50–249 employees] and large enterprises [250 or more employees] only add up to 2.3% (ISTAT, 2019). Thus, the motivation for investigating the export behaviour of smaller rather than larger manufacturers rides on the evident ubiquity of the former. Arguably, these smaller firms dominating the business landscape collectively bear more of the responsibility of delivering ‘made in Italy’ across the border. Not forgetting the crisis focus of this study, Italy was described as the ‘European epicentre of the outbreak during the first wave [of COVID-19]’ (Bourdin et al., 2021: 3). Also, Rapaccini et al. (2020: 225) believe that Italy is ‘undoubtedly the European country first and most extensively affected by the pandemic’. In a comparison of 13 European Countries, Gourinchas et al. (2020) found that Italian SMEs had the highest COVID-19 related failure rate at 22.49%. For these reasons, research into resilience and aspects of firm performance is promptly needed to generate insights for reversing Italian manufacturing SMEs’ vulnerability to COVID-19.

## Theoretical Background and Hypotheses Development

Firms’ appropriation of e-commerce evokes Prahalad and Hamel’s (1990: 82) notion of core competencies as ‘collective learning in the organisation, especially how to coordinate diverse production skills and integrate multiple streams of technologies’. Furthermore, the possession of assets exceeds the limits of the resource-based view because ‘it is not only the bundle of resources that matter, but the mechanisms by which firms learn and accumulate new skills and capabilities, and the forces that limit the rate and direction of this process’ (Teece et al., 1990: 11). To interrogate this line of thinking, the current conceptualisation contemplates organisational resilience as an attribute that possibly contributes to the rate of firms’ internationalisation as stimulated by e-commerce.

Moreover, to quote Burnard & Bhamra (2011: 5581), ‘organisations will experience disruptions and discontinuities. These disruptions can pose several threats to the incumbency of an organisation’. Particularly, with the borderless nature of contemporary risks (Smith & Fischbacher, 2009), such as the COVID-19 pandemic, the presence of unstable market conditions and the likelihood of organisational systems being disrupted has dramatically increased (Burnard & Bhamra, 2011). Steering high-impact and low probability events is now the order of business (Sheffi, 2007). To navigate these challenges, firms seek to adapt internal systems for the purpose of sustaining operations and cultivating competitive advantage (Sharma & Sharma, 2020), and this review considers e-commerce to be one such internal system.

To further address e-commerce, its prevalence has stemmed from widespread access to high-speed internet (Poggi et al., 2014). E-commerce leverages technologies such as mobile hardware, electronic fund transfer, digital supply chain and inventory management, electronic marketing, online transaction processing and automated data collection and interchange systems (Shahriari et al., 2015). In the last two decades, a rich stream of literature on manufacturing firms’ e-commerce adoption has accrued. For example, Lefebvre et al. (2005) categorised Canadian manufacturers into adopters and non-adopters by their rate of e-commerce uptake. They then contrived a logical evolutionary path for e-commerce penetration within these firms. Likewise, Alam et al. (2007) highlighted the role of relative advantage and compatibility in Malaysian manufacturer’ e-commerce venture. More recently, in Ghana, Ocloo et al. (2020) outlined perceived desirability, organisational readiness and competitive pressures as positive and significant harbingers of e-commerce adoption among local manufacturers. Rather than e-commerce, studies set in Italy (such as Cassetta et al., 2020; Rapacinni *et al*., 2020 and Giampietri & Trestini 2020) have more broadly investigated information communication technology [ICT] or digital technology [DT] adoption in non-manufacturing firms.

Nevertheless, Cassetta et al. (2020) draw parallels between DT in Italian SMEs’ supply chain and their export intensity. Export intensity is, by definition, firms’ export-to-sales ratio (Majocchi et al., 2005), and the most commonly operationalised measure of internationalisation behaviour (Wang & Ma, 2018). To this end, Hessels & Terjesen (2010) assert that, when internationalising, firms are faced with a decision of (1) exporting directly to overseas customers or (2) exporting indirectly through intermediaries. They add that, among other factors, firms’ access to knowledge and technology guides this decision-making in either way. To press the point, Gregory et al. (2019: 149) maintain that ‘specialised e-commerce marketing capabilities will lead to efficiencies in export marketing strategy and enhance export performance’. Yet, scholars examining this area have yet to make a distinction between direct and indirect exporting vis-à-vis the effect of e-commerce (Rialp-Criado & Komochkova, 2017; Motta, 2020). This is a telling empirical void, particularly for understanding e-commerce equipped manufacturers who may only produce semi-finished goods or parts that must first be sold to local intermediaries before they are exported.

Grounded in this background, this review now proceeds to hypothesis development.

### E-commerce and Direct Exporting

Since becoming commonplace, the association between e-commerce and export performance has drawn the attention of several studies (for example Gregory et al., 2007; Solaymani et al., 2012; Qi et al., 2020), owing to its [e-commerce] capacity to enhance consumption (Vithayathil & Choudhary, 2022). In fact, in less developed settings, e-commerce has been touted as a leveller of the export playing field that offers ‘producer firms new exchange mechanisms that enable them to compete on a more equal basis in world markets’ (Moodley and Morris, 2004: 155). In this respect, weighing evidence from manufacturers in Pakistan, Hussain et al., (2020) cite the importance of e-commerce in increasing exports and remedying the country’s balance of payments. Turning to Italy, alluding to digitalisation rather than e-commerce per se, Cassetta et al. (2016) hypothesised that the adoption of digital technologies influences the exporting behaviour of Italian manufacturers. Following this, the authors ascertained that firms ‘with front-end digital technologies are more likely to export’ (Cassetta et al., 2016: 305). Yet, to this point, there is no indication in prior research separating the link between e-commerce and direct versus indirect exports. To interrogate the likely disproportion in these outcomes, the first hypothesis is posed:

***H1***. *E-commerce is significantly and positively related to direct exporting*

### E-commerce and Indirect Exporting

As previously hinted, theoretical and empirical relations between e-commerce and indirect exporting have hardly been drawn ab initio. Frazier (1999) reasoned that specialised distribution channels that are highly dependent on intermediaries ought to shun e-commerce. Instead, Frazier (1999: 236) explained, ‘manufacturers using an exclusive or highly selective distribution intensity approach where local dealer investments are crucial should likely stay away from using the internet as a sales-distribution channel. When a product’s price varies considerably across global markets, limiting the internet’s scope appears wise’. This suggests that where disintermediation [the bypassing of intermediaries] is impossible, channel relationships need to be maintained offline and not compromised by e-commerce (Houghton & Winklhofer, 2004). Indeed, from manufacturers’ perspective, intermediaries are not only conveyor belts for exporting but warehouses where products can be deposited for a reasonable fixed cost (Ahn et al., 2011). Ahn et al. (2011: 75) add that ‘manufacturers face a trade-off between incurring a high fixed cost and directly exporting to a market and incurring a lower fixed cost to access a market through intermediaries. Ishii (2021) contends that the value of intermediaries depends on the degree of integration or independence in the export channel structure, to the extent that it decreases channel maintenance costs and yields export success. In anticipation of the degree to which e-commerce capacitates export intermediation among Italian manufacturers, it is expected that, in the COVID-19 context, the adoption of e-commerce increases SMEs’ visibility to local intermediaries. Hence, the second hypothesis is framed:

***H2***. *E-commerce is significantly and positively related to indirect exporting*

### E-commerce and Resilience

Increasingly, when faced with intense market competition, e-commerce is viewed by SMEs as one option in a suite of technologies that can enhance resilience (Gunasekaran et al., 2011). This has also proven true in the COVID-19 downturn. Recently, Prim & Sa (2020) ascertained that Brazilian SMEs incurred significantly lower impact on total sales if they expanded their e-commerce operations in response to the pandemic. Such evidence validates Hohenstein et al.’s (2015) portrayal of resilience as responding quickly to disruptions in an effort to return to an original situation or grow by moving to new, more desirable states that enhance customer service, market share and financial performance. Focusing on manufacturers, the challenges facing businesses relate to demand, supply, and control risks in the value chain (van Hoek, 2020). At the onset of COVID-19 movement controls, Rapaccini et al. (2020) found that managers in Northern Italy expected a double-digit decline in turnover that would only be redeemable by e-commerce sales. Certainly, in China, where economic recovery has already been attained, Zhan & Chen (2021) demonstrate how instrumental e-commerce has been especially for promoting and distributing perishable products in manufacturing subsectors such as food processing. Reardon et al. (2021) also attest to a spike in the rate of e-commerce diffusion in pursuit of resilience in Asian and Latin American supply chains. These indications prompt a third hypothesis:

***H3***. *E-commerce is significantly and positively related to resilience*

### Resilience and Direct Exporting

Extensive export activity is generally considered to be a signal of a thriving economy (Psycharis et al., 2014; Braese et al., 2019). There is also a dominant view that exporting firms are more likely to be resilient and exhibit superior crisis performance than non-exporters (Eppinger et al., 2018; Nakatani, 2017). In fact, resilience is key to SMEs as these firms are more vulnerable when unforeseen adverse events occur (Pal et al., 2014). To overcome their resource constraints, SMEs need to develop resilience to be competitive in international markets (Gunasekaran et al., 2011; Rahman & Mendy, 2019). Particularly, as international markets are associated with uncertainty and are more competitive than local markets, resilience is essential for SMEs to succeed especially in times of crisis (Lafuente et al., 2019). In effect, resilience helps SMEs to be more flexible, responsive, and competitive in international markets (Gunasekaran et al., 2011). On this basis, the following hypothesis is proposed:

***H4***. *Resilience is significantly and positively related to direct exporting*

### Resilience and Indirect Exporting

In the same manner that structural and relational social capital increase and sustain export intensity (Ling-Yee, 2004), it is presumptive that these factors may be a proxy for resilience that facilitate indirect exports through intermediaries during crises. Particularly, it has been observed that export intermediaries provide valuable information about foreign markets beyond the purview of small exporters (Kumar & Bergstrom, 2007). Accordingly, information asymmetry between trading parties that intensifies during economic crises can mainly be attenuated by the intelligence held and shared by intermediaries (Lu et al., 2017; Rostamkalaei 2017). In this sense, when information asymmetry is rife, exporters can benefit from intermediaries providing a further layer of communication between themselves and overseas customers (Oh, 2017). Likewise, Peng & York (2001: 327) maintain that the use of intermediaries ought to be weighed against ‘their possession of valuable, unique and hard to imitate resources which help minimise their clients’ transaction and agency costs’. On this note Suwannarat (2016) revealed that a key indicator of intermediaries’ performance is their capacity to reduce exporters’ transaction cost. The stance here is that intermediaries provide some of the social and relational capital that may be relied on by exporters during crises. To recall, Gaudenzi et al. (2021) also alluded to the value of e-commerce for managing information asymmetry and enabling logistical operations. Hence, the last hypothesis aims to test whether there is a positive association between being resilient by calling upon intermediary resources and the undertaking of indirect exports. Thus:

***H5***. *Resilience is significantly and positively related to indirect exporting*

## Method

### Data and Measures

The data for this study were obtained from the 2020 COVID-19 follow-up survey [Round 2] conducted in Italy by The World Bank (2021a). Recent studies by Williams & Kedir (2019) and Jha & Bose (2020) have also been based on World Bank data. From an initial panel of 760 firms, the data were reduced by four principles. First, only manufacturing firms were retained^1^. Second, firms with more than 250 employees were also removed for empirical interest in SMEs. Third, all cases with missing and ‘don’t know’ responses were removed to pre-empt statistical distortion and estimation bias. Fourth, only firms that were currently open with ongoing operations were selected. That said, there was a remainder of 249 SME manufacturers for analysis. Regarding the measures, there were five variables of (1) E-commerce (ECOMM), (2) Resilience (RESIL), (3) Indirect Exports (INDEXP), (4) Direct Exports (DIREXP) and Firm Size (SIZE). Table 1 outlines the items and measurement scale for each of these.

**Table 1:** Measurement Details

Variable	Item	Scale
ECOMM	Currently what is the share of this establishment’s online sales out of total sales?	Continuous
RESIL	Keeping the cost structure as it is now, how many weeks would this establishment be able to remain open if its sales stopped as of today?	Continuous
INDEXP	In the last completed month, what percentage of this establishment’s sales were indirect exports (sold domestically to third parties that export products)?	Continuous
DIREXP	In the last completed month, what percentage of this establishment’s sales were direct exports?	Continuous
SIZE	Permanent, full-time employees end of last month	Continuous

### Sample Characteristics

All 249 firms were small and medium manufacturing businesses with no more than 250 employees. In terms of size, 41% had up to 19 employees, 33.3% had between 20 and 99 employees, while 25.7% had 100 to 250 employees. Regarding country representation, representativeness is construed from the implementation report where The World Bank (2021b: 1) clarified that the COVID-19 ‘follow-up surveys re-contact all establishments sampled in the standard enterprise survey using stratified random sampling’. Table 2 gives a glimpse of the cases observed by firm size [number of employees].

**Table 2:** Sample Characteristics

Size	Frequency	Percent
0–19	102	41.0
20–99	83	33.3
100–250	64	25.7
**Total**	**249**	**100.0**

## Analysis

The hypothesis testing procedure was based on the robust path analysis algorithm (using stable 1 resampling method) in WarpPLS version 7.0 (Kock, 2020). This approach was considered relevant for this study as it allows the simultaneous testing of all variables in the model, including mediators (Kock & Gaskins, 2014). This approach has also been taken by other studies predicting internationalisation in various guises (Ammeer et al., 2021; Haddoud et al., 2021b).

### Measurement Model

Prior to undertaking path analysis, the reliability and validity of constructs need to be discerned. Nevertheless, the current variables are all single item indicators for which the calculation of composite reliability, Cronbach’s alpha and average variance extracted does not apply. Yet, to ensure that no collinearity issues would severely distort the findings, the variance inflation factor [VIF] of all variables was assessed. As shown in Table 3, all VIF scores did not exceed Hair et al.’s (2011) 5 threshold.

**Table 3:** Collinearity Diagnostic

	*ECOMM*	*RESIL*	*INDEXP*	*DIREXP*	*SIZE*
***VIF***	*1.031*	*1.113*	*1.179*	*1.210*	*1.192*

### Structural Model and Hypothesis Testing

**Fig. 1:**
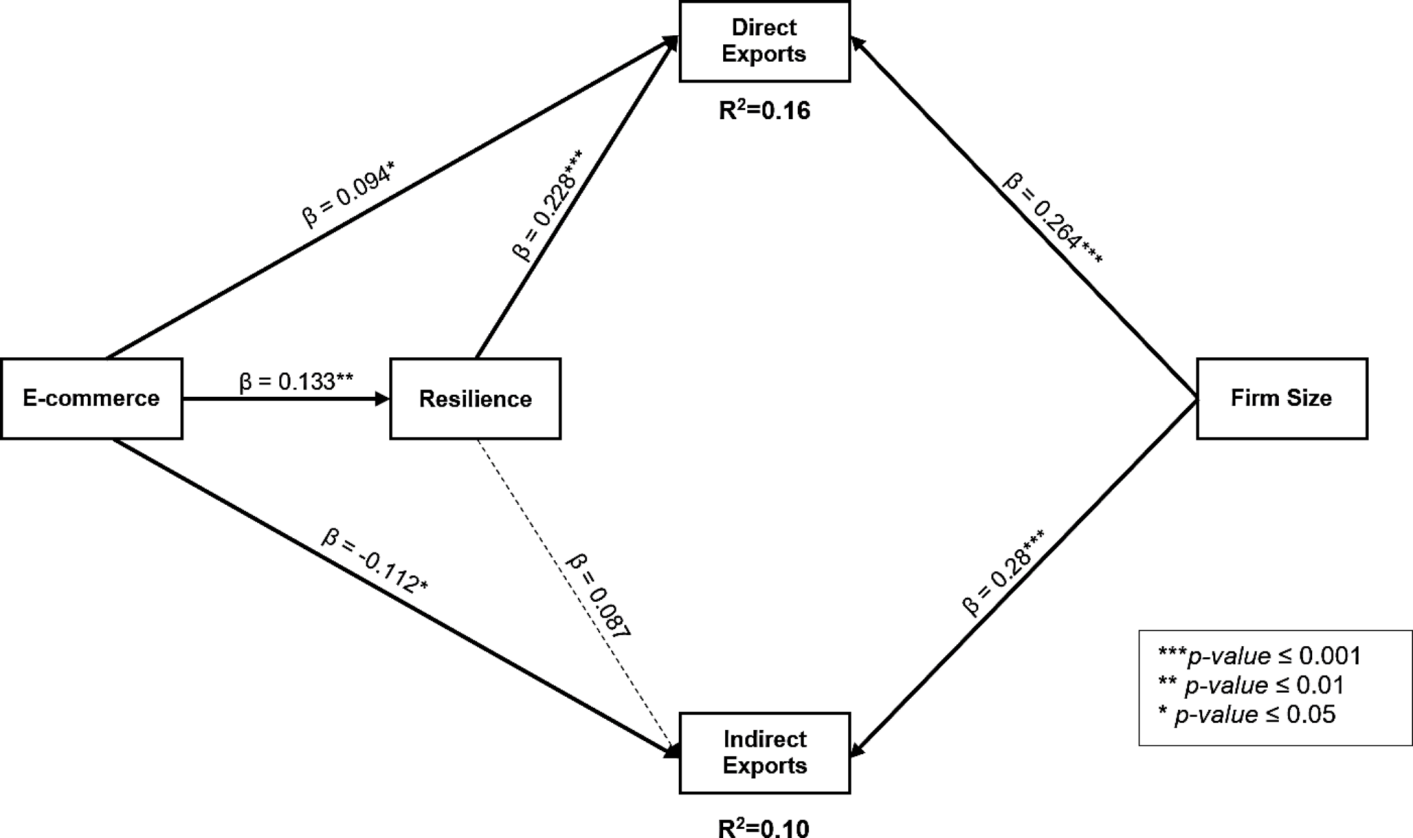
Structural Model

In Fig. 1 above, the inner model relationships are examined by interpretation of the path coefficients (β) and *p*-values.

To explain, in the first segment, the link between e-commerce and direct exports was positive and significant (β = 0.094*). Thus, H1 was supported. In contrast, e-commerce exhibited a significant but negative association with indirect exports (β = -0.112*), which did not support H2. Still in the first segment, e-commerce had a positive and direct association with resilience (β = 0.133**), which in turn increased direct exports (β = 0.228***) [supporting H3 and H4]. In contrast, resilience had a trivial association with indirect exports (β = 0.087), which did not provide sufficient support for H5. As for the indirect influence, the results failed to confirm a significant relationship linking e-commerce to direct and indirect exports [*p*-values for sums of indirect effects = 0.21 and 0.37 respectively]. Regarding firm size, having a higher number of employees appeared to be significantly associated with direct (β = 0.264***) and indirect exports (β = 0.28***). The structural model predicted 16% of direct exports and 10% of indirect exports for firms in the sample. Table 4 recaps these results.

**Table 4:** Hypothesis Testing

Hypothesised Relationship	Path Coefficient	*p*-Value
***H1***. E-COMMERCE ⇒ DIRECT EXPORTS	0.094	0.039
***H2***. E-COMMERCE ⇒ INDIRECT EXPORTS	-0.112	0.018
***H3***. E-COMMERCE ⇒ RESILIENCE	0.133	0.007
***H4***. RESILIENCE ⇒ DIRECT EXPORTS	0.228	< 0.001
***H5***. RESILIENCE ⇒ INDIRECT EXPORTS	0.087	0.051

## Discussion

The study advances extant works by uncovering the influence of Italian SMEs’ adoption of e-commerce on resilience and exporting activity (direct and indirect) in the context of COVID-19. In such times of crisis, SMEs’ resilience becomes paramount for survival and e-commerce is deemed a viable pathway and solution for firms to develop. Thus, the extant literature is awash with calls to uncover the influence of e-commerce in relation to the internet (Jean & Kim, 2020; Liu et al., 2020; Mathews et al., 2021), and the role of resilience (Lafuente et al., 2019; Rahman & Mendy, 2019) in enhancing SMEs’ internationalisation. Likewise, studies distinguishing between direct and indirect forms of exporting have also been called for (Rialp-Criado & Komochkova, 2017; Motta, 2020). The present findings have addressed these gaps.

Overall, the path results show that e-commerce is positively associated with direct exports and resilience. Moreover, resilience is positively related to direct exporting. The higher the SMEs’ share of online sales, the longer they remain in business in times of crisis. Likewise, resilient businesses were more likely to sell directly to international markets. However, resilience played a trivial role in stimulating indirect sales. Hence, the separation of direct and indirect exports moves the literature forward. The results are next discussed one at a time.

Firstly, the finding that online business activity boosts resilience and direct exports uphold recent evidence arguing that e-commerce helps companies remain in business amidst the COVID-19 crisis (Prim & Sa, 2020; Rapaccini et al., 2020; Zhan & Chen, 2021; Reardon et al., 2021), and boosts exports (Hussain et al., 2020). This positive influence can be explained through the prism of transaction costs. To illustrate, Abebe (2014) maintains that e-commerce enhances the efficiency of SMEs’ internal business processes and reduces transaction costs. This has been previously confirmed in the Italian context where SMEs with e-commerce facilities reduced distribution costs, enhanced their processes, and boosted profitability (Santarelli and D’Altri, 2003). Equally important, the adoption of e-commerce allows SMEs to generate key marketing data on consumer habits as well as supply chain information that can improve forecasting accuracy and reduce costs (Ramanathan et al., 2012). It is believed that such advantages are crucial in the context of a pandemic and more precisely during lockdowns as many SMEs with shoestring resources have had to migrate online to stay afloat (Hussain et al., 2021). Under the circumstances, cost reduction is needed to ensure survival when faced with declining sales and adaptation to buyers’ behaviour can be aided by dynamic data on consumer trends and more accurate supply chain forecasting. In a study investigating Italian firms following the 2008 financial crisis, Alonso and Bressan (2015) found that high/increasing cost was perceived to be by far the most serious threat facing businesses in the wine sector. Overall, it is argued that SMEs’ resilience necessitates a proactive approach towards gathering and utilising knowledge (Gunasekaran et al., 2011), and this can be achieved through online activity. In contrast, the adoption of e-commerce was negatively associated with indirect exporting. In this sense, it can be concluded that through e-commerce, SMEs are able to establish direct relationships with foreign customers, which exempts them from needing local intermediaries.

Secondly, SMEs’ resilience was positively associated with direct international sales. This result is consistent with previous evidence suggesting that exporters are typically more resilient than their non-exporting counterparts (Eppinger et al., 2018; Nakatani, 2017). In addition, Gunasekaran et al., (2011) and Rahman & Mendy (2019) stress that SMEs need to develop resilience to be competitive in international markets. Resilience is particularly relevant to SMEs as these firms are more vulnerable when unforeseen/adverse events occur (Pal et al., 2014). However, through resilience, SMEs can develop international flexibility, ambidexterity, and technological capabilities. International markets are known to harbour uncertainty and are more competitive than local markets to the extent that SMEs require resilience to thrive and re-enter, especially after previous failures and in times of crisis (Lafuente et al., 2019). As an attribute, resilience helps SMEs to be more adaptable, responsive, and competitive in global markets, which will eventually increase export performance (Gunasekaran et al., 2011).

Third, while the results validated the positive relationship between resilience and direct exporting, the influence on indirect exporting was trivial. To comprehend this result, it is important to recall the definition of indirect exporting theorised in this study. It was conceptualised that indirect exporting takes place when firms sell their products domestically to third parties for subsequent export. SMEs typically use indirect exporting to overcome knowledge gaps, avoid uncertainties and pass on inherent risks to foreign market intermediaries (Hessels & Terjesen, 2010). Motta (2020) explains that indirect exporting through intermediaries is an option for SMEs lacking appropriate resources to navigate challenges in export markets. According to Li (2004), intermediaries are well placed to perform certain export related functions more efficiently and less costly, hence helping SMEs to save costs (Peng & York, 2001). Rialp-Criado and Komochkova (2017) also recognise that, in comparison, indirect exporting is less intensive compared to direct exporting. Therefore, it could be argued that resilience is not needed in this instance since firms are not directly involved in international markets. Hence, attributes such as flexibility and ambidexterity may not be necessary.

Finally, the structural model also controlled for the effect of firm size on direct and indirect exporting. It was then determined that the higher the number of employees the greater the intensity of direct and indirect exporting. This finding endorses earlier cited contentions by Mittelstaedt et al. (2003) and Garmestani et al. (2006) that more employees enhance export related performance. This is also an important determination in the context of Italian manufacturers who are mostly small and micro enterprises. This study has found that labour intensity positively predicts export intensity in the specific context of small Italian manufacturers.

## Implications, Future Research and Limitations

To summarise, the verdict of this investigation is that e-commerce is a catalyst for resilience and direct exporting. In this regard, theoretical and practical implications are now outlined followed by limitations and prescribed avenues for future research.

### Implications

Beginning with the theoretical contributions, first, this study breaks new ground by investigating the links between e-commerce, resilience and the two forms of exporting. The resulting findings will apprise measurement and investigation of these variables in more national, sector and crisis contexts. Also, disentangling direct and indirect exports advances specificity in the distinctive effect of these relationships especially as the use of composite constructs has been found to cause underestimation of correlations (Bracken, 1996; Craven et al., 2003; O’Mara et al., 2006). The current structural model has effectively pre-empted this issue and, in one stroke, attended to Lafuente et al. (2019) and Rahman and Mendy’s (2019) exhortation for new evidence in the link between resilience and internationalisation. Second, this study offers a going concern conceptualisation of resilience. This new understanding offers a more definitive point-in-time estimation of firms’ probability to bounce forward by first considering organisational stamina. Third, as has yet to be undertaken in extant literature, the study has examined and reported evidence from the Italian manufacturing sector that is of interest to scholars (Rapaccini et al., 2020).

Turning to the practical implications, ‘between April and August 2020, Italian banks issued almost one million government guaranteed loans to around 900,000 small businesses for an aggregate amount of €79 billion’, in accordance with an emergency liquidity decree announced by the Italian government (Core & De Marco, 2021: 2). Therefore, SME owner/managers in the Italian manufacturing sector are encouraged to access available support and invest in e-commerce and staffing to increase their resilience and export performance. Matarazzo et al. (2021) recently confirmed that digital transformation programmes such as e-commerce significantly enhance the performance of Italian SMEs. By the same token, export promotion agencies like the Italian Trade Agency can reflect on these findings and recommend e-commerce capability and talent acquisition as a condition for offering support to SMEs. Such procedures will simultaneously nurture resilience and export behaviour. At the local level, other trade promotion organisations like the Italian Business Chambers can also reflect on the findings to offer timely counsel for optimising SMEs’ resilience and export intensity.

### Limitations and Future Research

Four shortcomings are acknowledged that may pave way for further research. In the first place, it is probable that other firm attributes excluded from the structural model, such as firm age and servitisation, also have a bearing on the degree of export intensity estimated. Future studies can account for these factors to present a fuller picture of the firm environment in connection with exporting. Other lines of inquiry into the effect of e-commerce and resilience on outcomes such as general performance and inventory turnover could be interrogated by forthcoming studies. Moreover, to build on the current findings, scholars may conduct qualitative studies based on interviews to understand the nature and features of e-commerce systems adopted by manufacturing SMEs in Italy. Secondly, the current inquiry is a single country and single sector study. New investigations are welcomed to validate the structural model in neighbouring countries with a comparable economic profile as well as in non-manufacturing settings. Third, the drawbacks of e-commerce have not been considered in this study. Obvious value is lost by firms from reduced customer interaction and feedback, and this will in turn have an adverse effect on resilience. An OECD report outlined that, for SMEs, despite the anticipated benefits, e-commerce is not without its challenges. These include greater associated costs, higher cyber risks, possible disruptions and potential conflicts of interests when dealing with online platforms and possible anti-competitive practices (OECD, 2021). Similarly, Sharma & Mukhopadhyay (2022) caution that e-commerce firms are typically targeted by cyber-hackers, making the prospect of incurring enormous financial loss a genuine possibility. Hence, going forward, scholars may contemplate these factors when examining manufacturing as well as retail and service sectors in Italy. Finally, owing to the cross-sectional nature of the data analysed, only association is inferred in the path diagram and not causation. Future studies can opt for a longitudinal or qualitative comparative approach that may prove causality in the model.

## Footnotes

1 These were extracted using the type of questionnaire completed by the firms (i.e. manufacturing, retail or other services).
